# Clinical profile of patients admitted in an acute psychiatric ward before and during the COVID-19 lockdown

**DOI:** 10.1192/j.eurpsy.2021.295

**Published:** 2021-08-13

**Authors:** A. Giménez Palomo, N. Baldaquí, L. Colomer, F. Gutiérrez, E. Pujal, P. Barrio, M. Bioque, E. Vieta, I. Pacchiarotti

**Affiliations:** Department Of Psychiatry And Psychology, University Of Barcelona, Hospital Clínic, Barcelona, Spain

**Keywords:** coronavirus, lockdown, Hospitalization, acute psychiatric ward

## Abstract

**Introduction:**

The COVID-19 pandemic entailed several changes in health and medical assistance, economy, and lifestyle. In the Acute Psychiatric Ward of the Hospital Clínic of Barcelona, the implementation of restrictive measures was necessary in order to ensure patients’ safety.

**Objectives:**

To compare clinical profiles and course of hospitalization of patients admitted before and during the COVID-19 lockdown in our Acute Psychiatric Ward.

**Methods:**

All patients admitted from January 7^th^ to February 25^th^ and from March 19^th^ to May 7^th^ of 2020 in the Acute Psychiatric Hospitalization Unit of Hospital Clínic of Barcelona, Spain, were retrospectively included for analysis and divided into two groups according to the period when they were admitted. Statistical analyses were performed using SPSS, 23.0 version.

**Results:**

A total of 117 inpatients were included (73 admitted before lockdown and 44 during lockdown), being 50.4% male, with a mean age of 42.4 (SD 15.73). Patients from the first group presented a significantly higher proportion of antidepressants prescription at discharge (p<0.05) and more substance use disorders (p<0.05). Regarding the lockdown group, 51% of patients manifested COVID-19-related stress. Time of hospitalization was significantly lower in the lockdown group (p<0.05), even though a significantly higher proportion of patients were discharged at home (p<0.05) compared with the first group.

**Conclusions:**

The situation of lockdown led to a series of changes in our unit and also in the profile of patients admitted, having shorter admissions, lower prescription of antidepressants, and often COVID-related stress. These differences should be considered in future situations in which restrictive measures may be necessary.

**Disclosure:**

No significant relationships.

